# Cordycepin Modulates Microglial M2 Polarization Coupled with Mitochondrial Metabolic Reprogramming by Targeting HKII and PDK2

**DOI:** 10.1002/advs.202304687

**Published:** 2024-06-18

**Authors:** Xin Zhong, Shiqiang Gong, Linghui Meng, Weifan Yao, Ke Du, Linchi Jiao, Guowei Ma, Jingwei Liang, Binbin Wei, Xin Jin, Junhui Tong, Jianru Dong, Mengyu Liu, Menglin Gao, Huachao Jia, Wenjuan Jiang, Zhihua Yu, Yanzhe Wang, Xiaohong Sun, Minjie Wei, Mingyan Liu

**Affiliations:** ^1^ School of Pharmacy China Medical University Shenyang Liaoning 110122 China; ^2^ Liaoning Medical Diagnosis and Treatment Center Shenyang Liaoning 11067 China; ^3^ He University Shenyang Liaoning 110163 China; ^4^ The First Affiliated Hospital of China Medical University Shenyang Liaoning 110002 China; ^5^ The Fourth Affiliated Hospital of China Medical University Shenyang Liaoning 110165 China; ^6^ Science Experiment Center China Medical University Shenyang Liaoning 110122 China

**Keywords:** cordycepin, HKII, metabolic reprogramming, microglial polarization, PDK2

## Abstract

The microenvironment mediated by the microglia (MG) M1/M2 phenotypic switch plays a decisive role in the neuronal fate and cognitive function of Alzheimer's disease (AD). However, the impact of metabolic reprogramming on microglial polarization and its underlying mechanism remains elusive. This study reveals that cordycepin improved cognitive function and memory in APP/PS1 mice, as well as attenuated neuronal damage by triggering MG‐M2 polarization and metabolic reprogramming characterized by increased OXPHOS and glycolysis, rather than directly protecting neurons. Simultaneously, cordycepin partially alleviates mitochondrial damage in microglia induced by inhibitors of OXPHOS and glycolysis, further promoting MG‐M2 transformation and increasing neuronal survival. Through confirmation of cordycepin distribution in the microglial mitochondria via mitochondrial isolation followed by HPLC‐MS/MS techniques, HKII and PDK2 are further identified as potential targets of cordycepin. By investigating the effects of HKII and PDK2 inhibitors, the mechanism through which cordycepin targeted HKII to elevate ECAR levels in the glycolysis pathway while targeting PDK2 to enhance OCR levels in PDH‐mediated OXPHOS pathway, thereby inducing MG‐M2 polarization, promoting neuronal survival and exerting an anti‐AD role is elucidated.

## Graphical abstract

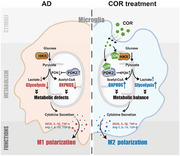

## 1 Introduction

Microglia (MG), as the primary immune monitor of the central nervous system (CNS), remains in a quiescent state under physiological conditions and serves the role of immune surveillance. Numerous studies have demonstrated that microglia can rapidly alter their morphology and phenotype to adopt an activated state in pathological conditions, existing broadly between two distinct states, namely a classical “pro‐inflammatory” (M1‐like) phenotype or an “alternative activation” (M2‐like) phenotype.^[^ ^1^ ^]^ Classically activated (M1‐like phenotype) microglia release pro‐inflammatory factors such as TNF‐α and IL‐1β to combat pathogens. Conversely, alternatively activated microglia (M2‐like phenotype) promote tissue repair and regeneration by releasing Arg‐1, IL‐10, and other factors to achieve neuroprotective effects.^[^ ^2^ ^]^ Throughout the progression of Alzheimer's disease (AD), microglia are continuously activated and recruited around amyloid‐β (Aβ) plaques, with their transition between different phenotypes regulating the neuronal microenvironment and determining the ultimate fate of neurons.^[^ ^3^ ^]^ Therefore, understanding the molecular mechanisms of neuronal microenvironment mediated by microglial M1/M2 phenotypic switch is crucial for developing new therapeutic strategies for AD.

Abnormalities in cerebral glucose metabolism are believed to precede Aβ deposition or phosphorylation of τ protein and have proven valuable for early diagnosis of AD.^[^ ^4^ ^]^ Various CNS disorders such as multiple sclerosis, Huntington's disease, and amyotrophic lateral sclerosis exhibit abnormal metabolic changes, indicating that alterations in energy metabolic characteristics significantly impact neural cell function and fate.^[^ ^5^ ^]^ As the energy biogenetic centers within cells, mitochondria provide the necessary energy for maintaining cellular homeostasis. Glucose is a primary energy source for CNS cells, and it is primarily metabolized through oxidative phosphorylation (OXPHOS) and glycolysis. Several metabolic enzymes involved in glucose metabolism, including hexokinase (HK), pyruvate dehydrogenase (PDH), and pyruvate dehydrogenase kinase (PDK) can influence cellular energy metabolism by finely regulating OXPHOS and glycolysis.^[^ ^6^ ^]^

Previous research has mainly focused on the changes and mechanisms of energy metabolic characteristics of neurons in the CNS.^[^ ^7^ ^]^ However, emerging evidence suggests that the energy metabolism in MG, rather than in neurons and astrocytes, plays a pivotal role in AD and permanent middle cerebral artery occlusion.^[^ ^8^ ^]^ Additionally, tumor‐associated macrophages with the same myeloid origin as MG have been suggested to exhibit distinct energy metabolic features in their different polarized phenotypes,^[^ ^9^ ^]^ indicating a potential linkage between energy metabolism and phenotypic transition. However, whether there is a causal relationship between alterations in energy metabolism and phenotypic switch in MG remains unclear. Therefore, an extensive exploration of the intrinsic connection between microglial energy metabolism and phenotypic transformation, as well as the underlying molecular mechanisms, will provide a new perspective for AD therapy and the development of candidate anti‐AD drugs.

Cordycepin (3′‐deoxyadenosine, COR) is the first nucleoside antibiotic isolated from fungi, exhibiting anti‐inflammatory, anti‐apoptosis, antioxidative, and immunoregulatory activities in cancer, leukemia, and diabetes.^[^ ^10^ ^]^ Our previous research has shown that COR plays a significant neuroprotective role and is associated with microglial polarization.^[^ ^11^ ^]^ However, it remains unknown whether metabolic reprogramming and its impact on microglial polarization are involved in the anti‐AD effects of COR. In this study, we have revealed that the metabolic reprogramming induced by HKII‐mediated glycolysis and PDK2‐mediated OXPHOS, coupled with MG‐M1 polarization, plays a crucial role in determining the fate of AD neurons. Furthermore, we have identified for the first time that COR is distributed into MG mitochondria using HPLC‐MS/MS combined with mitochondrial isolation. We also elucidated that as a potential novel compound for treating AD, COR promotes glycolysis and OXPHOS to drive metabolic reprogramming by dual‐targeting HKII and PDK2 while coupling with MG‐M2 polarization to exert anti‐AD effects. This offers a new perspective on understanding the relationship between metabolic reprogramming and phenotypic polarization in MG to modulate the neuronal microenvironment of AD, laying the foundation for designing and developing a novel drug targeting mitochondrial energy metabolism for AD treatment.

## 2 Results

### 2.1 COR Treatment Restores Mitochondrial Homeostasis of Activated Microglia to Ameliorate Learning and Memory Deficits in APP/PS1 Mice

**Figure 1:**
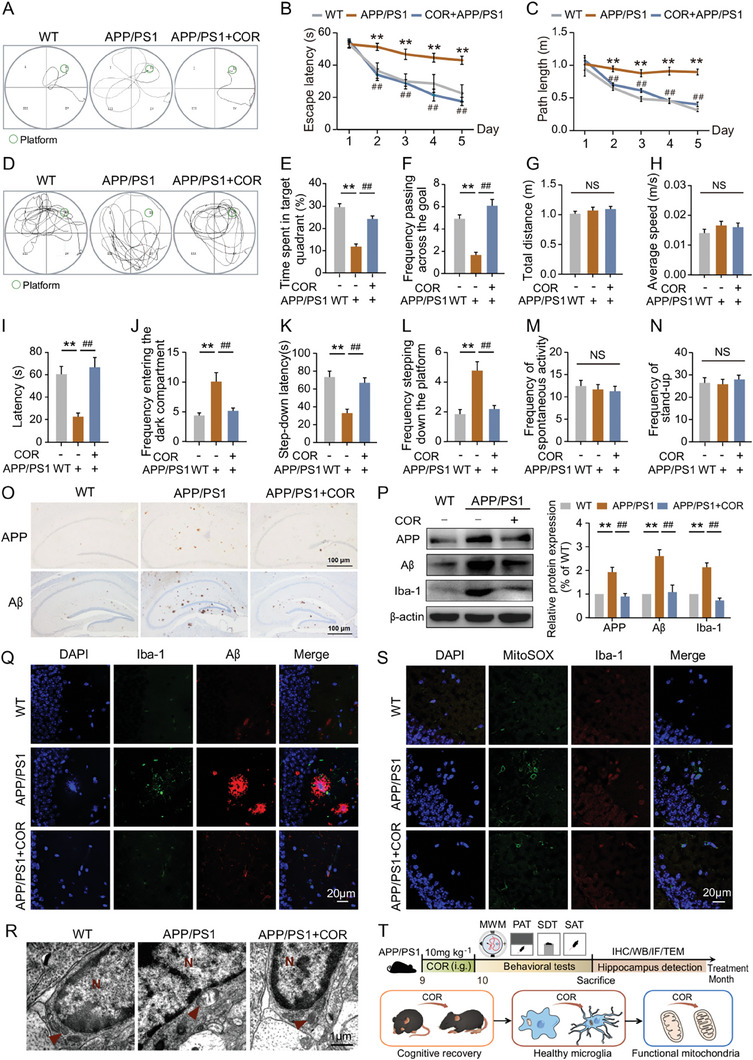
COR treatment restores mitochondrial homeostasis of activated microglia to ameliorate learning and memory deficits in APP/PS1 mice.

The learning and memory abilities of 9‐month‐old APP/PS1 mice were evaluated through behavioral experiments after a 4‐week administration of COR by gavage. In the navigation test of MWM, the escape latency and swimming distance of APP/PS1 mice were significantly longer compared to the WT group. Still, these deficits were markedly improved following COR treatment (**Figure** **1**A–C). During the probe trial, APP/PS1 mice exhibited a shorter stay in the target quadrant and passed through the original platform location fewer times than WT mice. However, treatment with COR resulted in increased time spent in the target quadrant and more crossings over the platform for APP/PS1 mice without affecting swimming distance or average speed (Figure 1D–H), which indicates an alleviation of spatial memory deficits. Additionally, in PAT and SDT, there was a notable increase in frequency and decrease in latency for entering the dark compartment exhibited by APP/PS1 mice compared to WT counterparts; these indicators were reversed with COR treatment (Figure 1I–L), suggesting an enhancement in memory consolidation. Furthermore, no significant differences were observed in autonomous activities among groups (Figure 1M,N), indicating that improvements in spatial learning and memory deficits can be attributed to COR rather than changes in locomotivity.

The hippocampal analysis further revealed that COR treatment ameliorated both APP and Aβ aggregation in APP/PS1 mice (Figure 1O). Western Blot validation confirms that COR inhibits the expressions of APP and Aβ, accompanied by a decline in Iba‐1 protein level in APP/PS1 mice (Figure 1P). Immunofluorescence staining results demonstrate a significant reduction of activated MG adjacent to Aβ plaques in the hippocampus of APP/PS1 mice after COR treatment (Figure 1Q).

The ultrastructure of hippocampal MG was examined to elucidate the beneficial effects of COR‐inhibited microglial overactivation on learning and memory performance in APP/PS1 mice. The results showed significant mitochondrial swelling and disappearance of the mitochondrial crest in APP/PS1 mice, which were alleviated following COR treatment (Figure 1R). Additionally, increased co‐expressions of Iba‐1 and mtROS were also rescued by COR treatment (Figure 1S). These results indicated that COR could maintain mitochondrial homeostasis, potentially contributing to the suppression of microglial activation and ultimately improving the learning and memory of APP/PS1 mice (Figure 1T).

In the navigation test of the Morris water maze (MWM, *n* = 12), A) the representative locus plot, B) escape latency, C) and path length of mice were shown for five consecutive days; D) In the probe trial of the MWM test (*n* = 12), the representative locus plot, E) time spent in the target quadrant, F) frequency passing across the goal, G) total distance and H) average swimming speed of mice were shown on the sixth day. In the Passive‐avoidance task (PAT, *n* = 12), I) latency and J) frequency of entering the dark compartment of mice were shown. In the Step‐down test (SDT, *n* = 12), the step‐down K) latency and L) frequency of stepping down the platform of mice were shown. In the Spontaneous activity test (SAT, *n* = 12) M) frequency of spontaneous activity and N) stand‐up of mice were shown. O) The APP and Aβ plaques were immunohistochemically stained in the hippocampus of mice (*n* = 3). P) Western Blot analyzed APP, Aβ, and Iba‐1 expression levels in hippocampal tissues of mice (*n* = 3). Q) The activated MG (Iba‐1) and Aβ plaques were co‐stained in the hippocampus of mice (*n* = 3). R) Representative images of ultrastructure in hippocampal MG of mice were obtained using TEM. S) Activated MG and mtROS were co‐stained in the hippocampus of mice (*n* = 3). T) The experimental timeline for behavior phenotypes followed by hippocampal detection in mice and schematic diagram for COR to exert an anti‐AD effect. *p < 0.05, **p < 0.01, compared with WT group; ^#^ *p *< 0.05, ^##^ *p *< 0.01, compared with APP/PS1 group. NS represents not significant. (Two‐way ANOVA followed by Tukey's multiple comparisons tests in (C, D) and one‐way ANOVA followed by Tukey's post hoc test in F to O). Data are presented as mean ± SEM.

### 2.2 COR Improves the Neuronal MicroEnvironment by Inducing a Shift in Microglial Polarization from the M1‐Like to M2‐Like Phenotype Rather Than Exerting Direct Neuroprotective Effects

**Figure 2:**
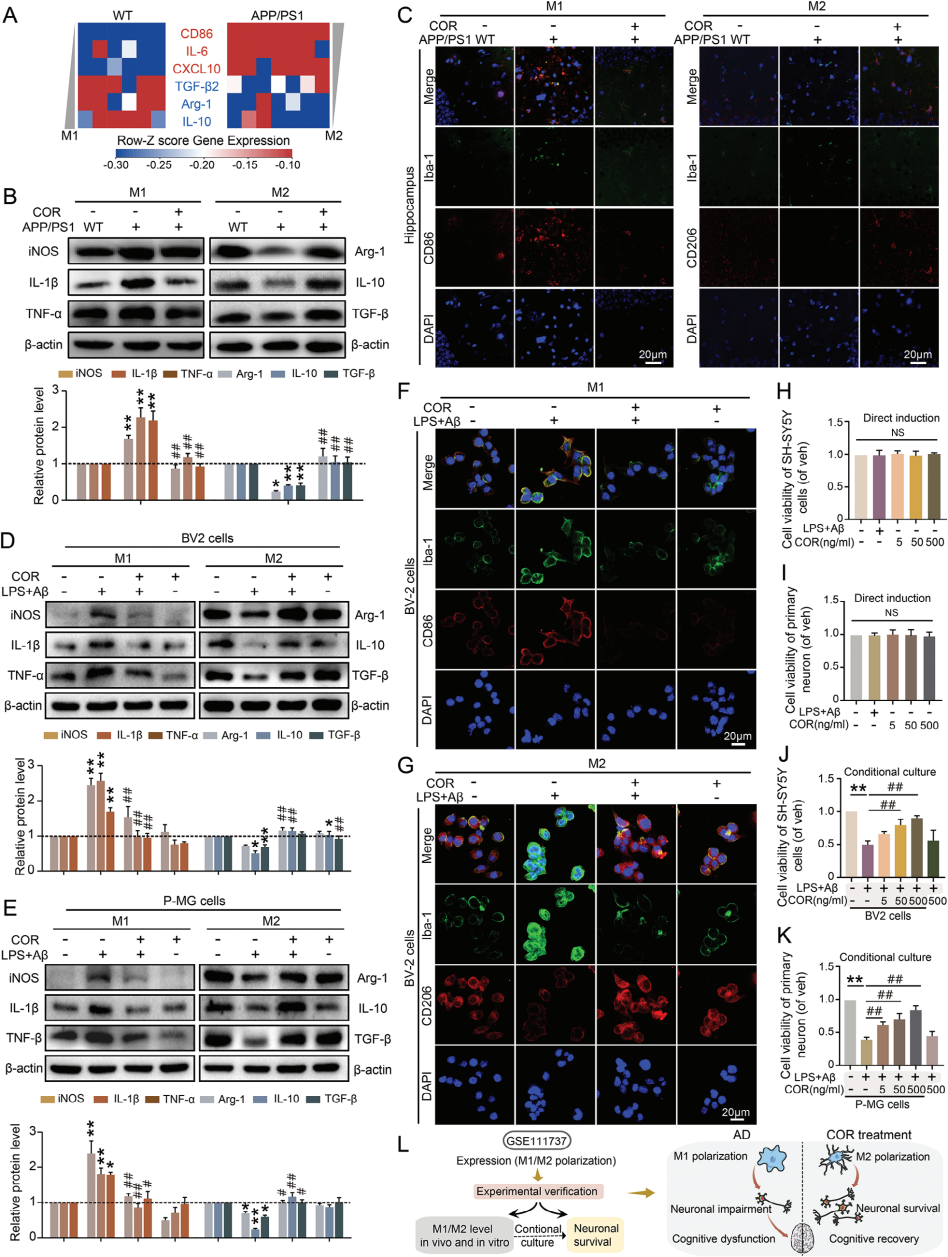
COR improves the neuronal micro‐environment by inducing a shift in microglial polarization from the M1‐like to M2‐like phenotype rather than exerting direct neuroprotective effects.

When MG are overactivated, their polarization state (M1‐like or M2‐like phenotype) will significantly impact the surrounding micro‐environment, which plays a crucial role in the progression of AD.^[^ ^12^ ^]^ Our analysis of the GSE111737 dataset revealed that genes associated with the M1 phenotype, such as CD86, IL‐6, and CXCL10, exhibited high expression levels in the hippocampus of APP/PS1 mice compared to those in the WT group. Conversely, genes related to the M2‐like phenotype, including TGF‐β2, Arg‐1, and IL‐10, were down‐regulated (**Figure** **2**A). Western Blot and immunofluorescence results further confirmed this. We observed increased expression levels of iNOS, IL‐1β, and TNF‐α and decreased expression levels of Arg‐1, IL‐10, and TGF‐β in the hippocampus of APP/PS1 mice compared to WT ones (Figure 2B). Furthermore, immunofluorescence showed a significant decrease in CD206 expression (a marker of M2‐like MG) accompanied by unregulated CD86 (a feature of M1‐like MG) in APP/PS1 mice along with Iba‐1 over‐expression (Figure 2C). Importantly, these changes were reversed after COR treatment (Figure2B,C), suggesting that COR treatment improved the cognitive deficits possibly by promoting microglial polarization from an M1‐like to an M2‐like phenotype. Additionally, BV2 cells and primary microglial cells stimulated by LPS+Aβ were employed to mimic the neuroinflammatory environment of AD. In both BV2 and primary MG, no significant alterations in the microglial polarization state were observed following COR treatment alone compared to the vehicle group. However, immunostaining and Western Blot analysis revealed that LPS+Aβ induced an increased M1‐like phenotype and a decreased M2‐like phenotype, which was subsequently recovered after COR treatment (Figure 2D–G). These findings indicated that COR treatment elicited anti‐AD effects possibly by promoting microglial M2 polarization.

Subsequently, we investigated whether the neuroprotective efficacy resulted from microglial M2 polarization induced by COR, or COR directly protected AD neurons. CCK8 results revealed that neither LPS+Aβ (LPS 1 µg mL^−1^ + Aβ 10 µmol) treatment nor COR treatment (5–500 ng mL^−1^) alone could affect neuronal viability, as shown in Figure 2H,I. Interestingly, the conditional media from LPS+Aβ‐induced BV2 cells and primary MG (P‐MG) exhibited significant toxicity toward SH‐SY5Y cells and primary neurons, suggesting that the M1 polarization of MG induced by LPS+Aβ was accountable for the reduced viability of neurons, rather than direct neurotoxicity. Furthermore, the conditional media from LPS+Aβ‐induced MG co‐treated with COR recovered the neuronal viability to baseline levels, whereas the direct addition of COR to neurons did not impact their survival when exposed to LPS+Aβ‐stimulated microglial conditioned media (Figure 2J,K). Thus, it was the neuronal micro‐environment mediated by microglial polarization that determined the fate of neurons, and COR exerted beneficial effects by suppressing microglial M1 and promoting M2 polarization to improve the neuronal micro‐environment rather than providing direct benefits to AD neurons (Figure 2L).

A) The gene expressions of M1/M2‐related cytokines in the hippocampus of WT and APP/PS1 mice were investigated by analyzing the GSE111737 using the Gene Expression Omnibus (GEO) query package (WT group: *n* = 6, APP/PS1 group: *n* = 7). B) Hippocampal lysates were subjected to Western Blot analysis for IL‐1β, iNOS, TNF‐α (M1‐like phenotype markers), and Arg‐1, IL‐10, TGF‐β (M2‐like phenotype markers) proteins (*n* = 3). C) Representative images of activated MG co‐staining with CD86 or CD206 in the hippocampus of mice. D, E) Immunoblot analysis cytokines associated with M1/M2 phenotype in BV2 and rat primary MG (P‐MG) (*n* = 3). F, G) Representative images of activated MG co‐staining with CD86 or CD206 in the BV2 cells. H, I) LPS+Aβ or COR (5–500 ng mL^−1^) directly treated SH‐SY5Y cells and rat primary neurons (P‐neuron), and the viability was detected by CCK‐8 assay (*n* = 3). J, K) The BV2 or P‐MG cells were exposed to LPS+Aβ and/or COR (5–500 ng mL^−1^), and their conditional media were subsequently transferred to SH‐SY5Y cells or P‐neurons for the evaluation of cell viability using CCK‐8 assay. Additionally, after the addition of conditioned media from LPS+Aβ‐stimulated MG to neurons, an additional 500 ng mL^−1^ of COR was directly administered to SH‐SY5Y cells or P‐neurons before assessing cell viability using the CCK‐8 assay (*n* = 3). L) The flowchart and schematic diagram illustrate that COR mediated microglial M2 polarization and played a neuroprotective role. *p < 0.05, **p < 0.01, compared with WT or Vehicle (Veh) group; ^#^ *p *< 0.05, ^##^ *p *< 0.01, compared with APP/PS1 or LPS+Aβ group (One‐way ANOVA, followed by Tukey's post hoc test). Data are presented as mean ± SEM.

### 2.3 Both OXPHOS and Glycolysis are Upregulated to Maintain Mitochondrial Homeostasis in AD Microglia Following COR Treatment

**Figure 3:**
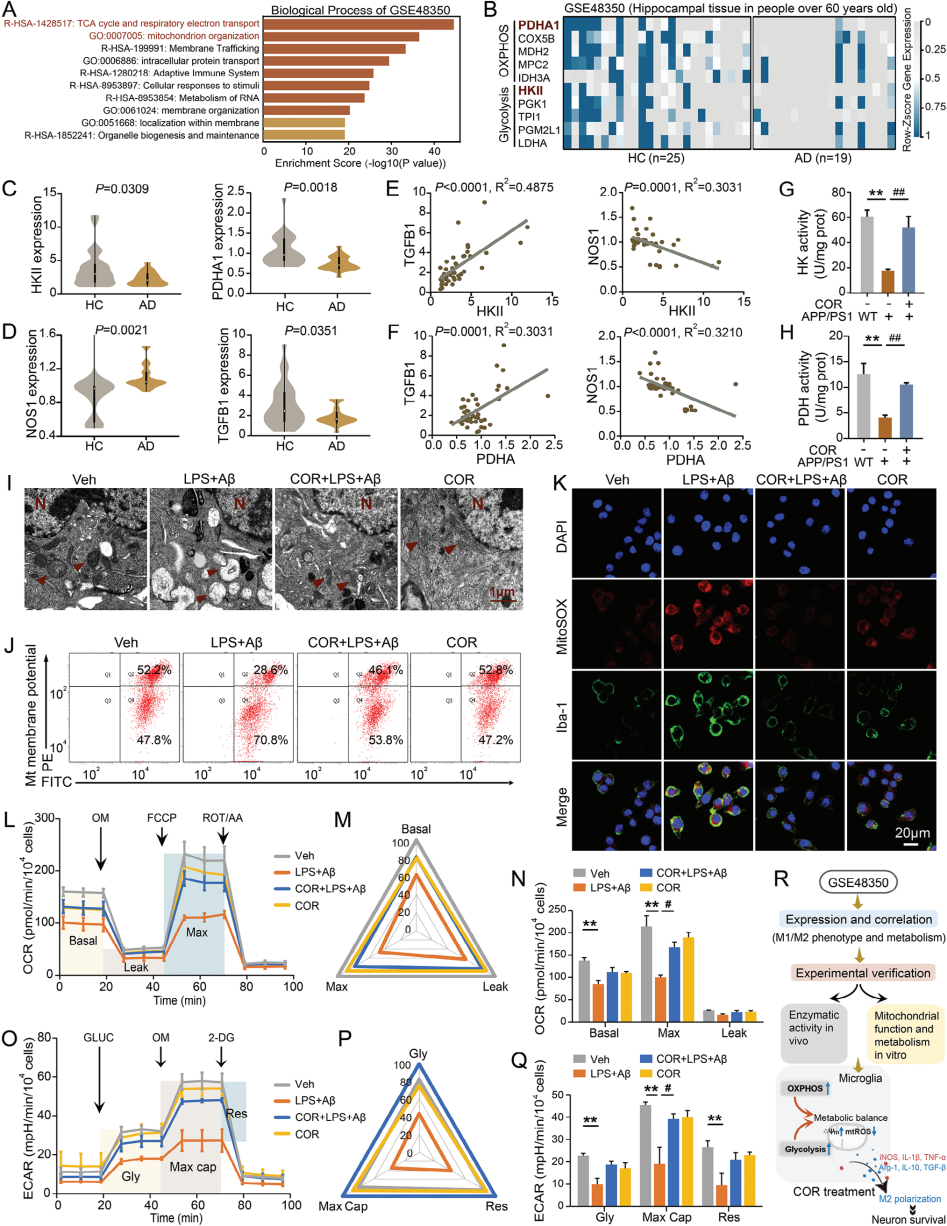
Both OXPHOS and glycolysis are upregulated to maintain mitochondrial homeostasis in AD microglia following COR treatment.

The potential mechanisms governing microglial polarization in AD were elucidated by retrieving matrix data from the GSE48350 dataset, followed by screening and importing the top 3000 differentially expressed genes (DEGs) into the Metascape database for enrichment analysis. Among the biological processes identified in GSE48350, the TCA cycle and respiratory electron transport, as well as mitochondrion organization, were recognized as the top two processes, indicating that mitochondrial function may be implicated in AD (**Figure** **3** **A**). Concurrently, heatmap clustering analysis revealed a significant decrease in the expression of differentially expressed genes (DEGs) related to these two processes in AD populations compared to healthy counterparts (Figure 3B). Successively, pyruvate dehydrogenase E1 component subunit alpha (PDHA1) and hexokinase II (HKII) were respectively marked as the most significant DEGs associated with oxidative phosphorylation (OXPHOS) and glycolysis process by analyzing the mitochondrial metabolic pathway in Reactome Pathway Database in GSE48350 (Figure 3B,C). To gain a deeper understanding of the correlation between the TCA cycle pathway and microglial polarization in AD, representative gene expressions and correlation analysis showed an increased level of M1‐related iNOS, along with reduced M2‐related TGFB1 gene expression in the AD group (Figure 3D). Furthermore, strong associations between PDHA1 and HKII, as well as iNOS and TGFB1, were respectively verified (Figure 3E,F), implying a potential involvement of disruption of mitochondrial metabolism and microglial M1 polarization in AD progression.

The hypothesis above was confirmed by observing decreased activities of HK and PDH in the hippocampus of APP/PS1 mice (Figure 3G,H). Subsequently, BV2 cells were utilized for in vitro validation. As anticipated, compared to the Vehicle (Veh) group, remarkable mitochondrial structural damage (Figure 3I), reduction in mitochondrial membrane potential (Figure 3J), and a boost in mitochondrial reactive oxygen species (mtROS) production (Figure 3K) were detected in LPS+Aβ‐induced BV2 cells, suggesting a potential impact of mitochondrial metabolism on AD pathogenesis. To further investigate the specific involvement of mitochondrial metabolism in AD, we analyzed the oxygen consumption rate (OCR) and extracellular acidification rate (ECAR) as indicators of cellular respiration and glycolysis, respectively. In comparison to the Veh group, LPS+Aβ stimulation resulted in a significant decrease in OCR level (Figure 3L), with a notable reduction in Basal respiration (Basal) and Maximal respiration (Max), as depicted in Figure 3M,N. Furthermore, ECAR levels also diminished in BV2 cells following LPS+Aβ treatment (Figure 3O), characterized by a marked reduction in Glycolysis (Gly), Maximal glycolytic capacity (Max cap), and Glycolytic reserve (Res), as shown in Figure 3P,Q. These results indicated that LPS+Aβ treatment induced severe disturbances in energy metabolism within microglial mitochondria. Subsequently, the combination of COR significantly alleviated microglial mitochondrial dysfunction, restoring OCR and ECAR levels and their corresponding parameters, including Max and Max cap. In contrast, COR alone did not elicit any changes in these parameters (Figure 3G–Q). These findings supported that metabolic instability based on OXPHOS and glycolysis might be a missing link between mitochondrial dysfunction and microglial polarization in AD. Furthermore, COR enhanced both OXPHOS and glycolysis to mitigate the mitochondrial metabolic disturbances, possibly contributing to its efficacy in promoting microglial M2 polarization (Figure 3R).

A) Pathway enrichment analysis of the top 3000 DEGs and their associated biological functions were conducted by analyzing the GSE48350 dataset using the GEO query package. B) A cluster heat map was generated to illustrate the differential expression of genes involved in the TCA cycle, respiratory electron transport, and mitochondrion organization processes between healthy controls (HC) and AD counterparts from the GSE48350 database (HC group: *n* = 25, AD group: *n* = 19). C, D) The gene expressions of HKII, PDHA1, NOS1, and TGFB1 were compared between HC and AD populations in the GSE48350 dataset. E, F) Linear correlation analysis was performed to examine the relationship between HKII, PDHA1, NOS1, and TGFB1. G, H) The activities of mitochondrial enzymes HK and PDH were measured in the hippocampus of WT mice, APP/PS1 mice as well as COR‐treated APP/PS1 mice (*n* = 3). BV2 cells were treated with or without LPS+Aβ and COR, I) ultrastructure of mitochondria among the groups was observed using TEM (*n* = 3); J) the mitochondrial membrane potential was examined using the JC‐1 method (*n* = 3); K) and the co‐staining for activated MG with mtROS was obtained (*n* = 3); L–N) OCR measurements displayed with quantification of basal respiration (Basal), maximal respiration (Max), proton leak (Leak) (*n* = 3); O–Q) ECAR measurements examined with quantification of glycolysis (Gly), maximal glycolytic capacity (Max cap), glycolytic reserve (Res) (*n* = 3). R) A flowchart describing how mitochondrial homeostasis is involved in AD progression via bioinformatics analysis followed by experimental verification and the regulation of COR. Oligomycin (OM); FCCP (Carbonyl cyanide 4‐(trifluoromethoxy)phenylhydrazone); Rotenone & Antimycin A (ROT/AA); 2‐deoxyglucose (2‐DG); Glucose (GLUC). *p < 0.05, **p < 0.01, compared with WT or Veh group; ^#^ *p *< 0.05, ^##^ *p *< 0.01, compared with APP/PS1 or LPS+Aβ group. (One‐way ANOVA, followed by Tukey's post hoc test). Data are presented as mean ± SEM.

### 2.4 COR Elicits Neuroprotective Efficacy via Inducing Microglial M2 Polarization Initiated by Mitochondrial Metabolic Reprogramming

**Figure 4:**
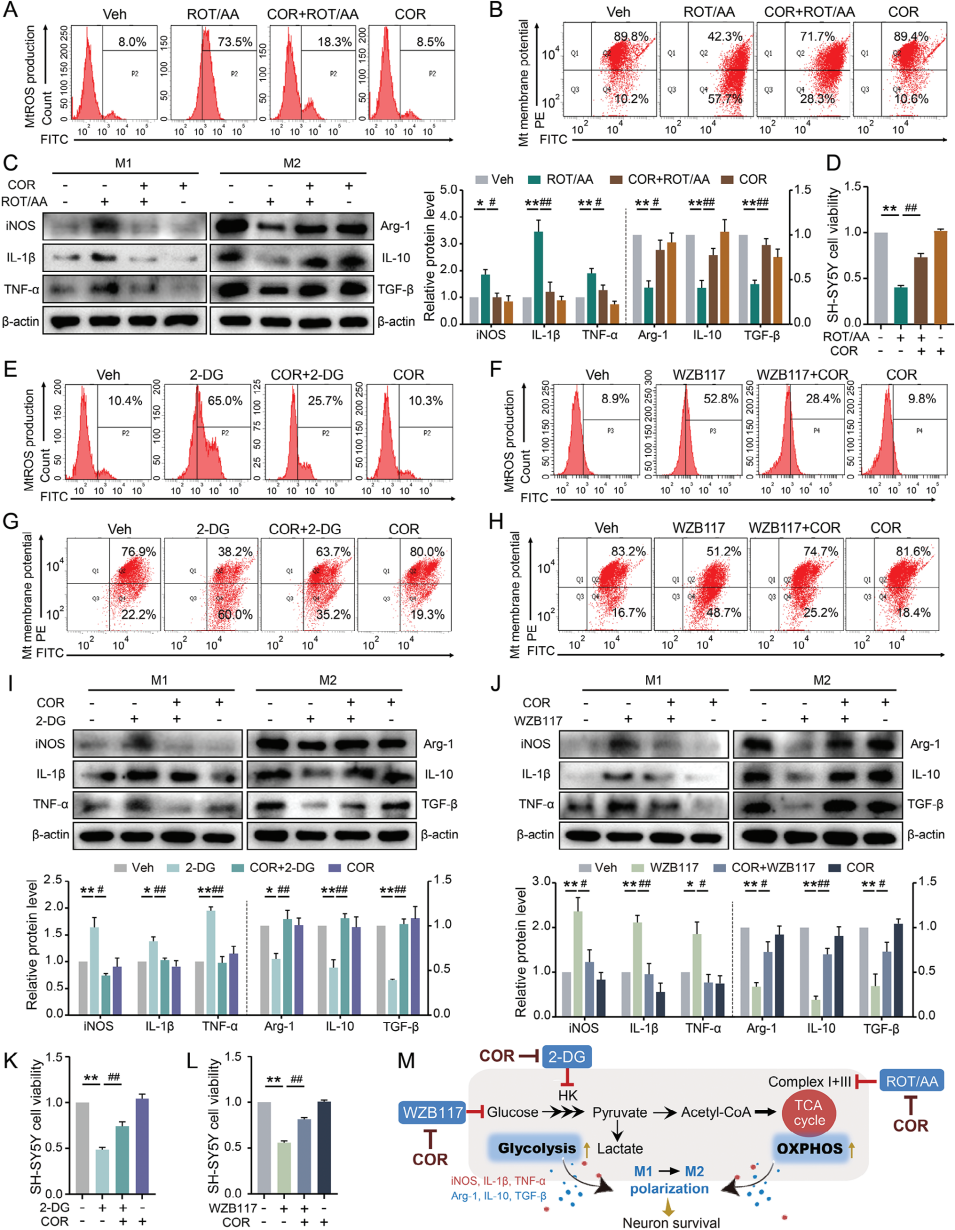
COR elicits neuroprotective efficacy via inducing microglial M2 polarization initiated by mitochondrial metabolic reprogramming.

As far as we are concerned, metabolic reprogramming is closely linked to microglial polarization.^[^ ^13^ ^]^ However, the chronological occurrence of these events remains unclear. This study utilized various inhibitors to disrupt OXPHOS and glycolysis in BV2 cells to investigate the interplay between metabolic reprogramming and microglial polarization. The respiratory chain inhibitor Rotenone & Antimycin A (ROT/AA) was employed to inhibit the TCA cycle. Our findings revealed that treatment with ROT/AA led to increased mtROS generation (**Figure** **4**A) and reduced mitochondrial membrane potential (Figure 4B) in BV2 cells. Concurrently, ROT/AA‐induced microglial M1 polarization in BV2 cells (Figure 4C), as mirrored by reduced expressions of M2 cytokines (iNOS, IL‐1β, TNF‐α) and elevated expressions of M1 cytokines (Arg‐1, IL‐10, TGF‐β). Furthermore, conditioned media obtained from BV2 cells after stimulation with ROT/AA resulted in decreased viability of SH‐SY5Y cells (Figure 4D). Co‐treatment with COR reversed ROT/AA‐induced mitochondrial dysfunction and polarized BV2 cells into M2‐like phenotype, thereby enhancing the viability of SH‐SY5Y cells. The COR treatment alone did not exhibit significant changes in the indicators above, suggesting that COR can preserve mitochondrial homeostasis by alleviating respiratory chain injury, thus promoting M2 polarization and enhancing neuron survival (Figure 4A–D). Subsequently, we respectively employed HK inhibitor 2‐DG and WZB117, a glucose transporter 1 inhibitor, to specifically block the glycolytic pathway, and assessed the impacts of COR on it.^[^ ^14^ ^]^ The results demonstrated that the effects of 2‐DG and WZB117 were in line with those obtained with ROT/AA (Figure 4E–L). Specifically, COR prevented mitochondrial dysfunction and induced polarization of BV2 cells toward an M2‐like phenotype in response to 2‐DG or WZB117. Additionally, it afforded neuroprotection against microglial conditional media induced by 2‐DG or WZB117 (Figure 4E–L). Based on our findings, we concluded that metabolic reprogramming serves as an initiator contributing at least partially to shifting MG from an M1‐like to an M2‐like phenotype which elicited neuroprotection (Figure 4M).

BV2 cells were treated with or without ROT/AA (0.5 µmol) and COR for 24 h, A, B) FACS plots showing representative mtROS expression levels and mitochondrial membrane potential in BV2 cells; C) Western blot analysis was applied to examine the cytokine expressions related to M1‐like or M2‐like phenotype; D) CCK‐8 assay was used to quantify the cell viability of SH‐SY5Y cells after treatment with conditioned media from BV2 cells. BV2 cells were treated with or without 2‐DG (50 mmol), WZB117 (10 µmol), and COR for 24 h, E–H) FACS plots showing representative mtROS expression levels and mitochondrial membrane potential in BV2 cells; I, J) Western blot analysis was applied to examine the cytokine expressions related to M1‐like or M2‐like phenotype; K, L) CCK‐8 assay was used to quantify the cell viability of SH‐SY5Y cells after treatment with conditioned media from BV2 cells. M) The schematic diagram of the action mode of ROT/AA, 2‐DG, or WZB117 on OXPHOS and glycolysis pathways and the regulation of COR. *p < 0.05, **p < 0.01, compared with Veh group; ^#^ *p *< 0.05, ^##^ *p *< 0.01, compared with ROT/AA or 2‐DG group. (One‐way ANOVA, followed by Tukey's post hoc test). Data are presented as mean ± SEM.

### 2.5 COR Specifically Targets HKII and PDK2 within the Mitochondria of MG to Regulate Mitochondrial Metabolism

**Figure 5:**
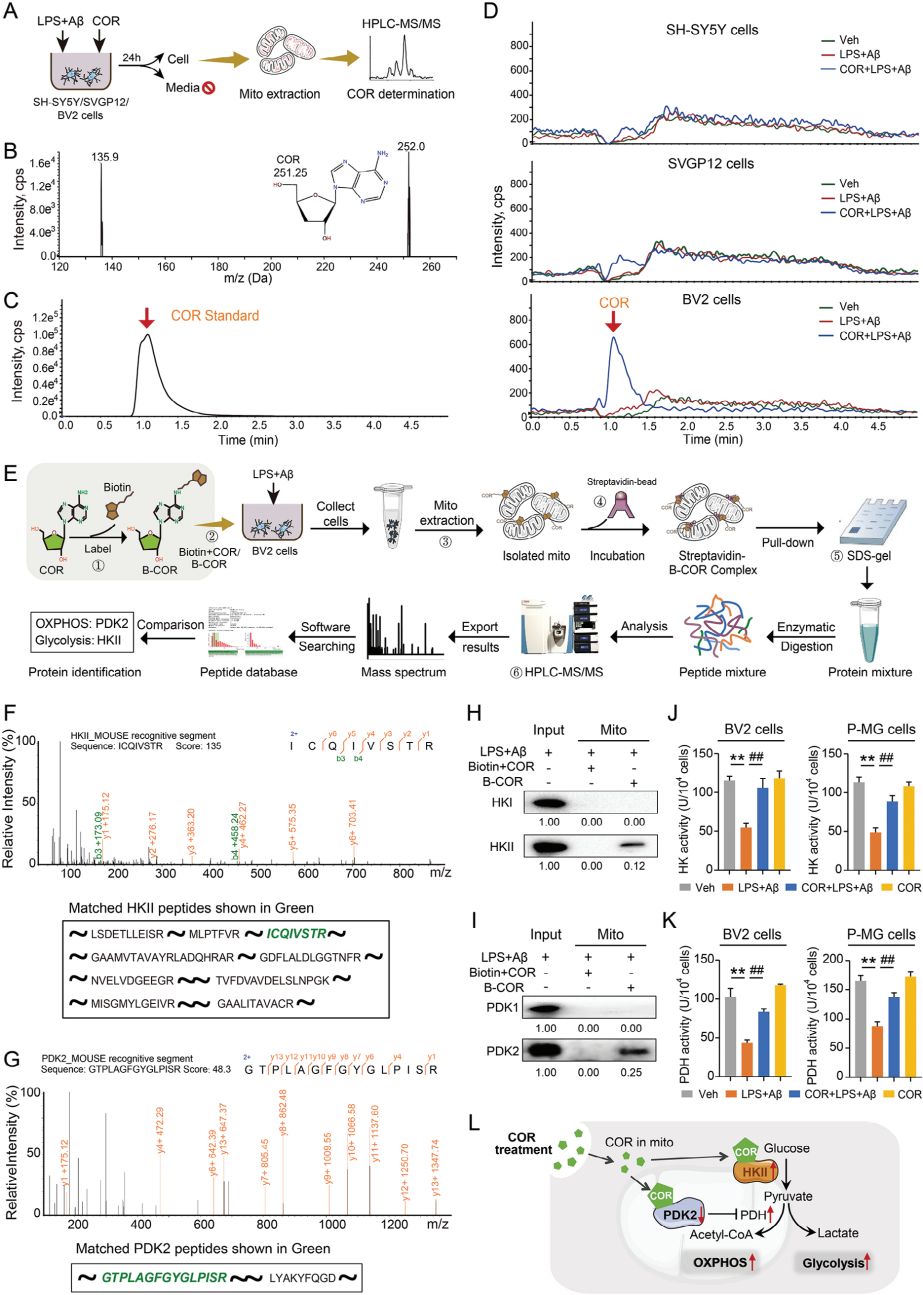
COR specifically targets HKII and PDK2 within the mitochondria of MG to regulate mitochondrial metabolism.

To investigate the role of COR in regulating metabolic reprogramming in AD, we isolated mitochondria and conducted a component analysis using HPLC‐MS/MS (**Figure** **5**A). Subsequently, COR was detected within the mitochondria of LPS+Aβ‐induced BV2 cells, rather than SH‐SY5Y cells and SVGP12 cells (Figure 5B–D), indicating its ability to distribute in the microglial mitochondria and potentially regulate metabolic reprogramming through specific interactions. We then synthesized a biotinylated‐COR (B‐COR) by chemically labeling COR with biotin. Mitochondria isolated from B‐COR or biotin+COR‐treated BV2 cells were incubated with streptavidin magnetic beads to enrich protein complexes for analysis using protein mass spectrometry and SDS‐PAGE (Figure 5E). Protein mass spectrometry revealed hexokinase II (HKII) and pyruvate dehydrogenase kinase 2 (PDK2) as the most enriched putative targets associated with glycolysis and OXPHOS respectively, which are unique to B‐COR treatment (Figure 5F,G). Further protein electrophoresis with streptavidin‐B‐COR complex showed that B‐COR‐precipitated lysates were bound to HKII and PDK2, rather than HKI and PDK1 (Figure 5H,I), which indicated that HKII and PDK2 were specific targets of COR, contributing to the regulation of high levels of OXPHOS and glycolysis, respectively. Moreover, significant decreases in LPS+Aβ‐induced BV2 and P‐MG cells’ HK and PDH activity were restored by COR treatment (Figure 5J,K). In conclusion, by dual‐targeting HKII and PDK2 in microglial mitochondria, COR enhances the activities of HK and PDH, leading to elevated levels of glycolysis and OXPHOS, which induces metabolic reprogramming (Figure 5L).

A) Flowchart depicting the determination of COR in isolated mitochondria from LPS+Aβ‐induced SH‐SY5Y cells, SVGP12 cells, and BV2 cells. B, C) Representative chromatograms, chemical structures, and full scan product ion spectra of COR standard (3 µg mL^−1^). D) Chromatograms show the presence of COR in isolated mitochondria from SH‐SY5Y cells, SVGP12 cells, or BV2 cells within the experimental groups. E) ① A biotinylated derivative of COR (B‐COR) was synthesized; ② B‐COR alone or biotin+COR (unbiotinylated) was incubated with LPS+Aβ‐induced BV2 cells; ③ their respective mitochondria were then isolated; ④ incubate with magnetic beads coated with streptavidin; ⑤ an enriched fraction of the protein complex was pulled down for SDS‐PAGE analysis; ⑥ subsequently, protein mass spectrometry was utilized to identify potential coprecipitating proteins that interact with B‐COR in isolated mitochondria. F, G) Representative chromatograms of the recognizable segments and sequence identities of HKII and PDK2 proteins. H, I) Protein electrophoresis using mitochondrial lysates was conducted to confirm the binding of the targets to the streptavidin‐B‐COR complex. J, K) The activities of HK and PDH in LPS+Aβ‐induced BV2 cells and rat primary MG were investigated. L) The schematic diagram described COR entering microglial mitochondria and interacting with HKII and PDK2 to promote OXPHOS and glycolytic pathways. *p < 0.05, **p < 0.01, compared with Veh group; ^#^ *p *< 0.05, ^##^ *p *< 0.01, compared with LPS+Aβ group. (One‐way ANOVA, followed by Tukey's post hoc test). Data are presented as mean ± SEM.

### 2.6 COR Enhances Mitochondrial Glycolysis to Induce Microglial M2 Polarization by Targeting HKII, Thereby Promoting Neuronal Survival

**Figure 6:**
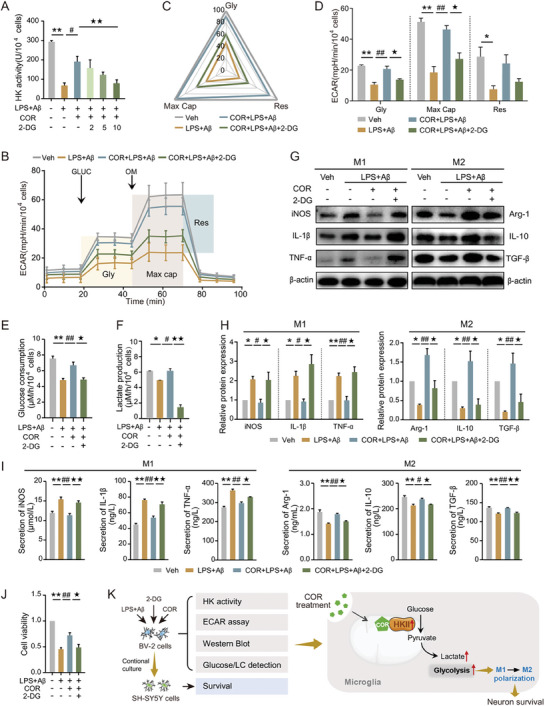
COR enhances mitochondrial glycolysis to induce microglial M2 polarization by targeting HKII, thereby promoting neuronal survival.

The initial crucial step in glucose metabolism is catalyzed by HK, which facilitates ATP‐dependent glucose phosphorylation to produce glucose‐6‐phosphate. BV2 cells were treated with 2‐deoxy‐d‐glucose (2‐DG), an HK inhibitor, to further investigate the interaction between COR and HKII on metabolic reprogramming and microglial polarization. Our results showed that 2‐DG could concentration‐dependently decrease the HK activity upregulated by COR, with the most significant inhibitory effect observed at a concentration of 10 mmol (**Figure** **6**A). Additionally, 2‐DG also suppressed the increased ECAR level (Figure 6B–D), glucose uptake (Figure 6E), and extracellular lactate secretion (Figure 6F) rescued by COR in LPS+Aβ‐induced BV2 cells, which indicated that reduced HK activity weakened COR's enhancement of glycolysis from glucose consumption to lactate production. As expected, 2‐DG shifted COR‐induced M2 polarization toward the M1‐like phenotype (Figure 6G–I) and decreased SH‐SY5Y cell viability when cultured with conditioned media from BV2 cells (Figure 6J). In conclusion, we concluded that COR enhanced the glycolytic levels through interaction with HKII, thereby promoting microglial M2 polarization and neuron survival in AD (Figure 6K).

BV2 cells were treated with or without COR, LPS+Aβ, and 2‐DG (2, 5,10 mmol) for 24 h. A) HK activity was investigated. B, D) ECAR was analyzed using the seahorse instrument, and glycolysis (Gly), glycolytic capacity (Max cap), and glycolytic reserve (Res) were quantified. E, F) Glucose consumption and extracellular lactate production were analyzed using the colorimetric method. G–I) Western Blot and ELISA were applied to determine the expression and secretion levels of IL‐1β, iNOS, TNF‐α and Arg‐1, IL‐10, and TGF‐β. J) CCK‐8 assay was used to investigate the cell viability of SH‐SY5Y cells exposed to conditioned media generated from BV2 cells. K) The schematic diagram described the interaction manner of COR and HKII regulating microglial polarization to elicit neuroprotective efficacy. *p < 0.05, **p < 0.01, compared with Veh group; ^#^ *p *< 0.05, ^##^ *p *< 0.01, compared with LPS+Aβ group; ^★^ *p *< 0.05, ^★★^ *p *< 0.01, compared with LPS+Aβ+COR group. (One‐way ANOVA, followed by Tukey's post hoc test). Data are presented as mean ± SEM.

### 2.7 COR Treatment Promotes OXPHOS by Targeting PDK2, Inducing Microglial M2 Polarization and Neuronal Survival

**Figure 7:**
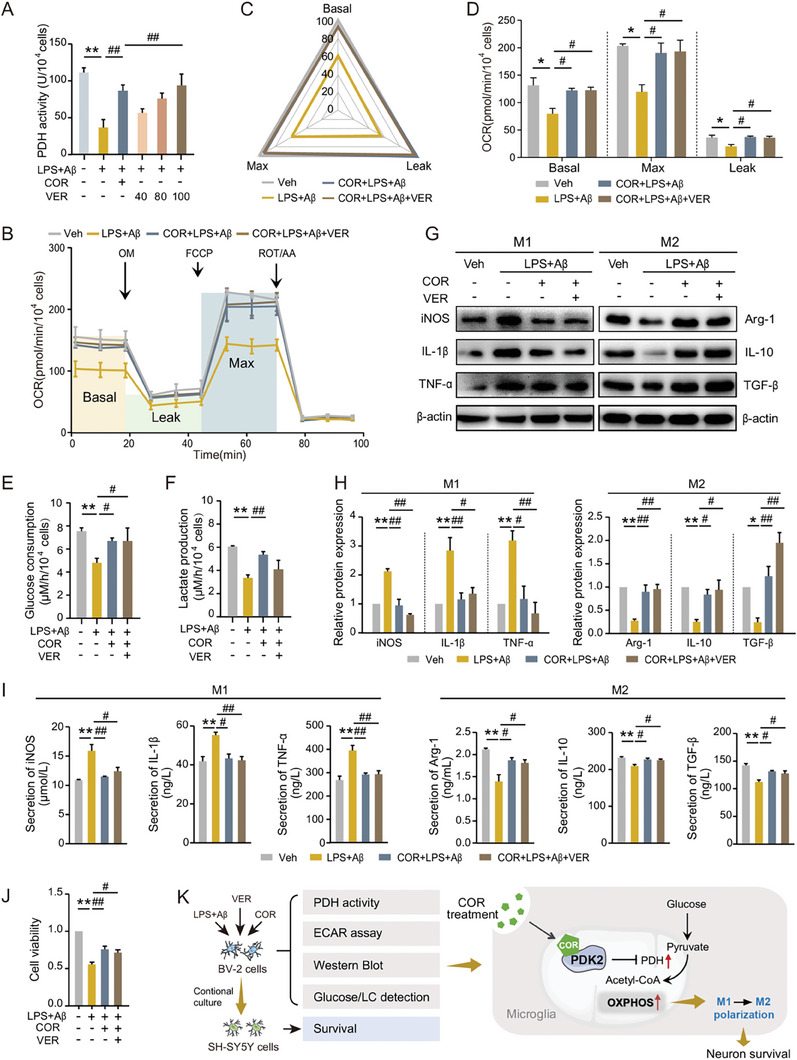
COR treatment promotes OXPHOS by targeting PDK2, inducing microglial M2 polarization and neuronal survival.

The simultaneous upregulation of OCR levels in LPS+Aβ‐induced BV2 cells by COR treatment prompted us to investigate the interaction between COR and PDK2 in mitochondrial function. As is well known, PDK inhibits the activity of pyruvate dehydrogenase (PDH) by phosphorylating the E1 subunit PDHA1, thereby suppressing aerobic respiration. Therefore, we investigated whether PDK2 could regulate OXPHOS by treating BV2 cells with the PDK inhibitor VER‐246608 (VER). The results demonstrated that VER rescued the decreased PDH activity induced by LPS+Aβ at a concentration of 100 nmol, similar to COR (**Figure** **7**A). However, compared to the LPS+Aβ group, VER treatment restored the OCR level (Figure 7B–D) and glucose consumption (Figure 7E), rather than extracellular lactate secretion (Figure 7F). These implied that VER could only increase PDH‐mediated OXPHOS levels by inhibiting PDK activity, but did not affect lactic acid production in the glycolysis pathway.

To further investigate the impact of PDK2 on COR's neuroprotective role, we found that VER induced polarization of BV2 cells toward the M2‐like phenotype (Figure 7G–I) and enhanced the survival of SH‐SY5Y cells cultured with conditioned media from LPS+Aβ‐induced BV2 cells (Figure 7J). These findings indicated that COR might interact with PDK2 in a manner similar to VER, thereby enhancing aerobic respiration and exerting an anti‐AD role (Figure 7K).

BV2 cells were treated with or without COR, LPS+Aβ, and VER‐246608 (VER) (40, 80, 100 nmol) for 24 h. A) PDH activity was investigated. B–D) OCR was analyzed using the seahorse instrument and basal respiration (Basal), maximal respiration (Max), and proton leak (Leak) were quantified. E, F) Glucose consumption and extracellular lactate production were analyzed using the colorimetric method. G–I) Western Blot and ELISA were applied to determine the expression and secretion levels of IL‐1β, iNOS, TNF‐α and Arg‐1, IL‐10, and TGF‐β. J) CCK‐8 assay was used to investigate the cell viability of SH‐SY5Y cells exposed to conditioned media generated from BV2 cells. K) The schematic diagram described the interaction mode of COR and PDK2 regulating microglial polarization to elicit neuroprotective efficacy. *p < 0.05, **p < 0.01, compared with Veh group; ^#^ *p *< 0.05, ^##^ *p *< 0.01, compared with LPS+Aβ group. (One‐way ANOVA, followed by Tukey's post hoc test). Data are presented as mean ± SEM.

**Figure 8:**
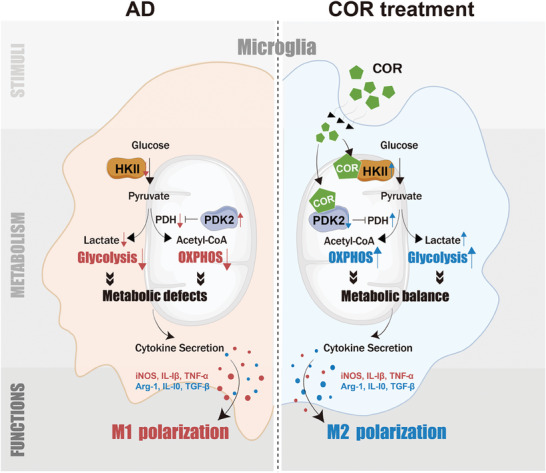
COR drove microglial M2 polarization coupled with mitochondrial metabolic reprogramming by dual‐targeting HKII and PDK2 to exert an anti‐AD effect.

In conclusion, our investigation has revealed that inhibition of glycolysis and OXPHOS metabolic pathways leads to an imbalance in mitochondrial homeostasis coupled with microglial M1 polarization and subsequent neuron loss in AD. Furthermore, the novel anti‐AD candidate COR enhances both glycolysis and OXPHOS to rescue abnormal metabolic reprogramming by targeting HKII and PDK2, inducing a shift in microglial phenotype from an M1 to M2‐dominant phenotype and ultimately promoting neuron survival (**Figure** **8**). These findings uncover a potential coupling mechanism between mitochondrial metabolic reprogramming and microglial polarization in AD, offering an innovative development strategy for new drugs targeting mitochondrial dysfunction and neuronal microenvironment.

The microglial mitochondrial energy metabolism in AD is characterized by reduced glycolysis and OXPHOS, leading to a coupling effect with MG‐M1 polarization and neuronal damage. COR is distributed in microglial mitochondria and binds to HKII and PDK2, thereby enhancing glycolysis and OXPHOS coupled with MG‐M2 polarization to improve the neuronal microenvironment following COR treatment.

## 3 Discussion

In this study, we have confirmed that the imbalance of energy metabolism resulting from the inhibition of glycolysis and OXPHOS in MG, along with mitochondrial dysfunction, triggers microglial M1 polarization, exacerbating the neuronal microenvironment and ultimately leading to neuron damage in AD. Additionally, we have discovered that COR, a potential anti‐AD candidate, improves learning memory function in 9‐month‐old APP/PS1 mice. Interestingly, COR did not directly protect neurons but instead enhanced the neuronal microenvironment by promoting glycolysis and OXPHOS to induce microglial polarization from M1‐like to M2‐like phenotype, thereby exerting anti‐AD effects. Mechanistically, COR distributes to mitochondria in BV‐2 cells induced by LPS+Aβ, increases ECAR levels and lactate production in the glycolytic pathway by targeting HKII while upregulating OCR levels in PDH‐mediated OXPHOS pathway by targeting PDK2, thus inducing microglial M2 polarization and contributing to AD neuronal survival.

The role of MG in the CNS is well established to be similar to that of peripheral macrophages, and their activation is heterogeneous. Recent studies have identified various AD‐related microglial phenotypes, including those involved in surveillance, pro‐inflammatory, anti‐inflammatory functions, and others.^[^ ^15^ ^]^ The response of MG to CNS diseases, such as acute brain injury, has been demonstrated to involve the activation and production of classical M1‐like (pro‐inflammatory) or alternatively activated M2‐like (anti‐inflammatory) phenotypes.^[^ ^1^ ^]^ Therefore, to facilitate the conceptualization of microglial activity characteristics, many studies have utilized these two phenotypes for classification purposes. They exert cytotoxic or anti‐inflammatory effects through M1/M2 polarization under different stimuli, which is a key factor affecting brain development and neuronal microenvironment.^[^ ^16^ ^]^ In the present study, we also confirmed the critical role of neuronal inflammatory microenvironment mediated by abnormally activated MG polarized to M1‐like phenotype in exacerbating AD neuronal cytotoxicity and cognitive impairment. Importantly, our findings demonstrate that the conditioned culture media of MG‐M1 phenotype could induce neuronal damage, rather than the direct toxic effects of LPS+Aβ itself on neurons, showing a distinction from previous research emphasizing direct neuronal toxicity.^[^ ^17^ ^]^ These findings underscore the significance of MG‐M1/M2 phenotypic transition‐mediated neuronal microenvironment in AD.

Metabolic disorders, such as reduced glucose utilization and mitochondrial dysfunction, are common characteristics of AD.^[^ ^18^ ^]^ Emerging evidence indicates that MG activation is associated with early alterations in energy metabolism in the blood and cerebrospinal fluid of AD patients.^[^ ^19^ ^]^ Furthermore, hypometabolism in the brain of 3xTg‐AD mice is associated with high expression of MG marker Iba‐1, rather than astrocyte marker GFAP,^[^ ^20^ ^]^ suggesting that impaired MG energy metabolism plays a pivotal role in AD. Venkataraman et al. also reported that the dysfunction of mitochondria and synapses at the early stage of AD, as detected by positron emission tomography (PET), correlated with AD patients' learning and memory impairments.^[^ ^21^ ^]^ In contrast to previous findings indicating a metabolic shift from OXPHOS to glycolysis in LPS‐induced BV2 cells,^[^ ^22^ ^]^ our study revealed that LPS+Aβ‐induced BV2 cells, polarizing toward M1‐like phenotype, exhibited a metabolic disorder characterized by inhibition of both glycolysis and OXPHOS. This provides an essential basis for understanding the imbalance of microglial energy reprogramming during AD progression. The discrepancy between these studies may be attributed to the different induction conditions for MG. LPS is more inclined to induce an inflammatory response in early AD, possibly leading to compensatory glycolysis effects. However, combined induction with LPS and Aβ mimics the late‐stage AD environment where MG metabolism becomes fully imbalanced and compensatory glycolysis effects are compromised, significantly reducing both OXPHOS and glycolysis. Importantly, we not only observed changes in MG energy metabolism induced by LPS+Aβ but also demonstrated that interference with aerobic phosphorylation or glycolysis using ROT/AA or 2‐DG occurred as an event prior to phenotypic transition and neuronal injury. This coupling effect between energy metabolic reprogramming and microglial polarization regulates the neuronal microenvironment and influences the fate of neurons affected by AD.

Additionally, we have identified a potential anti‐AD compound COR, and validated its neuroprotective effects in both in vitro and in vivo settings. Of particular significance is the discovery that the neuroprotective effect of COR is not attributed to its direct protection of AD neurons, but rather to its ability to shift microglial polarization from M1‐like to M2‐like phenotype, thereby promoting the survival of AD neurons and ameliorating behavioral deficits. This represents a novel finding that lays the groundwork for developing new anti‐AD medications to improve the neuronal microenvironment. Furthermore, we demonstrate for the first time that COR promotes mitochondrial energy metabolism through simultaneous upregulation of OXPHOS and glycolysis to maintain mitochondrial homeostasis coupled with MG‐M2 polarization, ultimately exerting neuroprotective effects. These findings differ from previous reports indicating that sodium rutin enhanced Aβ elimination and alleviated AD‐like symptoms by upregulating OXPHOS and inhibiting glycolysis.^[^ ^23^ ^]^ Our candidate compound COR enhances OXPHOS and significantly boosts glycolysis, highlighting its advantages in driving mitochondrial metabolic reprogramming leading to MG‐M2 polarization.

Based on our observation that COR induced microglial metabolic reprogramming, we focused on investigating COR's distribution within mitochondria. As anticipated, HPLC‐MS/MS combined with mitochondrial isolation revealed the presence of COR within microglial mitochondria for the first time, providing a mechanistic basis for understanding how COR exerts its effects at this organelle level. This discovery is consistent with our previous findings regarding microglial metabolic regulation by COR. In addition, Zhang et al. reported that flavonoid oroxylin A's antitumor effect on hepatocellular carcinoma and breast cancer was linked to its localization within tumor cell nuclei.^[^ ^24^ ^]^ Similarly, Reiter et al.^[^ ^25^ ^]^ demonstrated melatonin's ability to enter mitochondria and target them to exert antioxidant effects. However, these studies were limited to confirming compound distribution within target organelles without delving into their detailed mechanisms.

In the mitochondria, energy metabolism is regulated by a series of enzymes that modulate glycolysis and OXPHOS. Analysis of GEO microarray data in humans and mice revealed decreased HK activity in the brain, leukocytes, and microvasculature of AD patients, as well as in the hippocampus of mice.^[^ ^26^ ^]^ As a crucial metabolic enzyme involved in glucose metabolism and the glycolytic pathway, HK activity is positively correlated with glycolytic capacity. However, HK kinetic activity is inhibited when there is an increase in Adenosine diphosphate (ADP) concentration.^[^ ^27^ ^]^ Furthermore, Aβ triggered detachment of HKI from neuronal mitochondria, contributing to oxidative stress and neurodegeneration in AD.^[^ ^28^ ^]^ Recent research has shown that deletion of HKII in MG effectively promotes phagocytosis of Aβ plaques and mitigates cognitive impairment in 5xFAD AD mice.^[^ ^29^ ^]^ The involvement of HK in COR‐modulated glycolysis remains uncertain. The evidence supporting the relationship between COR and HKII includes our initial discovery that biotin‐labeled COR (B‐COR) fished out HKII from the mitochondria of MG, which was validated by protein spectrometry. In mammals, HKI and HKII are located at the mitochondrial outer membrane where they can preferentially acquire ATP produced by the mitochondria.^[^ ^30^ ^]^ Subsequent pull‐down assays confirmed that it was explicitly HKII (not HKI) bound to COR, and their combination enhanced HKII activity, which aligns with our previous findings showing an increase in ECAR and related glycolytic indicator Max Cap. Additionally, treatment with 2‐DG, an inhibitor for HK, reversed the effects of COR on levels of glycolysis and lactate production, MG‐M2 polarization, and neuronal survival. These results further confirmed that COR played a neuroprotective role by targeting HKII to regulate glycolysis coupled with a microglial switch to the M1‐like phenotype.

After clarifying the mechanism by which COR regulates glycolysis, our focus shifted to identifying specific targets for regulating OXPHOS within the mitochondria of MG. We discovered that PDK2, a key enzyme involved in OXPHOS, interacted with COR during B‐COR fishing experiments to identify potentially interacting proteins within MG. Subsequent pull‐down experiments confirmed that PDK2 (not PDK1) was specifically binding to COR. As A key enzyme in aerobic metabolism, PDK2 inhibits the activity of PDH, thus preventing pyruvate conversion into acetyl‐CoA entering the tricarboxylic acid cycle for OXPHOS. Previously only increased PDK1 expression was found in the frontal cortex of 6‐month‐old APP/PS1 mice,^[^ ^31^ ^]^ while overexpression of PDK1 in rat neuronal cells B12 exhibited reduced OCR.^[^ ^32^ ^]^ However, the relationship between PDK2 and MG polarization in AD has not been fully elucidated. The PDK2 inhibitor VER, showed similar efficacy to COR in enhancing PDH activity, promoting MG polarization and neuronal survival. This study showed that COR exerted neuroprotective effects by targeting PDK2 to upregulate PDH activity, thereby increasing OCR, promoting glucose utilization, and driving MG‐M2 polarization. Notably, VER increased OCR levels and glucose consumption without promoting lactate production as COR did, which may be due to COR's interaction with HKII, which could increase lactate production by promoting the glycolytic pathway. In contrast, VER might divert pyruvate away from the glycolytic pathway toward aerobic respiration, inhibiting the increase in lactate levels. Therefore, increased aerobic metabolism compensated for its inhibition of the glycolytic pathway, and an overall equilibrium of energy metabolism levels was achieved so that VER did not antagonize their coupling effects on MG phenotypic switch and the following benefits.

However, several intriguing questions remain unanswered: i) What are the precise interaction mechanisms between COR and HKII? Further molecular docking results from MOE revealed a high degree of conformational similarity between COR and ADP at the active site of HKII protein (data not shown), suggesting that COR may enter MG mitochondria to bind with specific HKII and occupy the position of the original substrate ADP, thereby relieving kinetic inhibition of HKII by ADP and increasing glycolysis levels. ii) What is the specific action of COR and PDK2? Similarly, we found COR could be inserted into a non‐competitive inhibitor pocket of PDK2 protein binding to VER (data not shown), suggesting that COR may bind to PDK2 in a manner similar to VER, thereby decreasing its activity and relieving its inhibitory effect on PDH activity, and triggering an increase in PDH‐mediated aerobic metabolism. However, these hypotheses require further discussion and verification in subsequent experiments.

In conclusion, this study has uncovered the critical role of imbalanced microglial metabolic reprogramming, characterized by reduced glycolysis and OXPHOS, in driving MG‐M1 polarization in AD neuronal fate. Additionally, COR exerted an anti‐AD effect by mediating mitochondrial metabolic reprogramming through dual‐targeting HKII/PDK2, leading to MG‐M2 polarization and improvement of the neuronal microenvironment. This study offers new insights into understanding the correlation between metabolic reprogramming and microglial phenotypic transformation, as well as the neuroprotective effect of COR, which lays an experimental foundation for advancing drug design and research targeting energy metabolism in AD.

## 4 Experimental Section

### Experimental Animals

The APP/PS1 (APPswe/PSEN1dE9)] double‐transgenic mice were obtained from the Jackson Laboratory, and the C57BL/6 mice and the SD rats were from the Laboratory Animal Center of China Medical University. The mice were in the SPF animal room, where there was a 12 h light/dark cycle at temperatures 20–25°C and relative humidity of 40–60%. Then, animals were allowed free access to food and water.

### Drug Administrations

9‐month‐old APP/PS1 mice were randomly assigned into two groups: the APP/PS1 group (*n* = 12) and the COR group (*n* = 12). Age‐matched C57BL/6 littermates were designated as the Wild Type (WT) group (*n* = 12). Cordycepin (10 mg kg^−1^ day^−1^; MedChemExpress) was intragastrically received in the COR group. Simultaneously, the corresponding volume of double distilled water was administered in the WT and APP/PS1 groups by gavage once daily for four weeks. All experimental animal procedures strictly complied with the Standard Medical Laboratory Animals' Care and Use Protocols Laboratory and the Animal Ethical Standards of China Medical University (SYXK(Liao)2013‐0007).

After the treatment, behavioral experiments were performed. All mice were then sacrificed, and the hippocampal tissues were homogenized and fixed for Western Blot and immunofluorescent staining and transmission electron microscope analysis, respectively.

### Morris Water Maze (MWM)

The MWM was operated to evaluate the effect of cordycepin on the spatial learning and memory performance of mice based on the previous study with minor modifications.^[^ ^33^ ^]^ Briefly, the MWM consisted of a circular pool, a platform that could be moved and hidden beneath the water surface, and an automatic image acquisition and processing system. In the navigation test, mice were put into the water with their heads facing the wall of the pool at four starting positions in the east, west, south, and north, respectively, and the time between the animals being put into the water and finding the platform was recorded. The escape latencies and path lengths from entering the water to reaching the platform were recorded for five consecutive days. In the probe trial test, after removing the platform on the sixth day, the mice were placed in water on the opposite side of the quadrant where the platform was located. The time spent in the target quadrant, the frequencies passing across the original platform location, the total distance, and the average swimming speed of mice within 60 s were recorded.

### Passive‐Avoidance Task (PAT)

PAT is a fear‐aggravated test for evaluating learning and memory in mice, which was implemented after MWM to measure memory retention. The method of operation was derived from previous studies.^[^ ^11^ ^]^ In short, PAT is divided into two stages: the training period and the formal test. Before the training, the mice were put into a bright room for 3 min to adapt to the environment. Once the mice entered the dark room, they were electrically shocked (voltage 36 V, 5 min), which made them return to the bright room. In the formal test 24 h later, latencies and frequencies that the mice entered the dark room within 5 min were recorded.

### Step‐Down Passive Avoidance Test (SDT)

The SDT was performed to assess the effects of cordycepin on the nonspatial memory performance of mice. Initially, mice were set on an insulated platform and adapted to the surrounding environment for 3 min. When the mice stepped down the platform, they immediately received an electric shock and jumped onto the insulated platform. The next day after training, the test session was carried out following the same procedure, and the step‐down latencies and the number of errors were recorded.

### Spontaneous Activity Test (SAT)

SAT was applied to test the effects of cordycepin on spontaneous locomotor activity. Briefly, mice were habituated in the autonomous movement system for 10 min, and the locomotivity and the frequencies of stand‐up were counted.

### Experimental Cell Lines

Mouse microglial BV2 cell line, human astroglioma SVGP12 cell line, and human neuroblastoma SH‐SY5Y cell line were obtained from the Institute of Basic Medical Sciences of the Chinese Academy of Medical Sciences. Cells were cultured in a humidified chamber with 5% CO_2_ at 37 °C. All cell culture plates were purchased from Jet Biofil.

### Isolation and Culture of Primary MG

The Sprague–Dawley (SD) rats within postnatal 24 h were used to prepare primary MG as previously described.^[^ ^34^ ^]^ Briefly, the hippocampus from the neonatal brain was isolated and digested using 0.125% trypsin. After centrifugation at 1500 rpm for 5 min, cell precipitates were re‐suspended with DMEM/F12 medium and filtered with a 70 µm cell strainer. Cells were counted and seeded at 2 × 10^5^ cells on l‐Poly‐Lysine‐coated culture flasks with 10 mL culture medium (DMEM/F12 medium containing 20% fetal bovine serum and 1% penicillin–streptomycin). 7–9 days later, the culture flask was tapped until the upper layer cells fell off. Purified microglial cells could be harvested after seeding and culturing for ≈3–5 days.

### Isolation and Culture of Primary Neuron

Consistent with the isolation process of primary MG, the hippocampus from newborn SD rats within 24 h was collected and digested (0.125% trypsin and DNA enzyme) to prepare the single‐cell suspension. After centrifuging at 1000 rpm for 5 min, the precipitation was mixed with culture media (DMEM/F12 medium containing 10% FBS, 1% Penicillin–Streptomycin–Gentamicin (P/S/G; Solarbio LIFE SCIENCES), and 1% horse serum) and seeded in l‐Poly‐Lysine‐coated culture flask. 2 h later, culture media were discarded and replaced with the Neurobasal medium (Gibco) containing 2% B27 (Gibco) and 100×P/S/G. Medium was changed every three days for about a week, the primary neuron could be used in experiments.

### Conditional Culture

For the collection of conditioned media, BV2 and rat primary microglial cells were pretreated with or without COR (500 ng mL^−1^) for 1 h before being stimulated with LPS (1 µg mL^−1^; Sigma–Aldrich) for 1 h and Aβ1‐42 (10 µmol; Sigma–Aldrich) for 24 h. Fresh culture media was added and incubated for a further 24 h period. The conditioned media was collected and centrifuged to remove the cells. The SH‐SY5Y or primary neuronal cells were seeded in 96‐well plates and incubated overnight. Then, the cells were treated with the conditioned media for 24 h.

### Immunofluorescent Staining and Confocal Microscope Observation

BV2 and rat primary microglial cells were seeded in confocal microscope dishes at 10^4^ cells per well and incubated overnight. The following day, cells were treated with cordycepin to a final concentration of 1 µg mL^−1^ for 1 h in the COR group. Then, the cells in the LPS+Aβ group and COR group were added LPS (1 µg mL^−1^) for 1 h and Aβ1–42 (10 µmol) for 24 h. The supernatants were removed, and the cells were fixed with 4% paraformaldehyde and permeabilized with 0.5% Triton X‐100 for 10 min. Subsequently, the cells were immunostained as previously described using anti‐Iba‐1 (Wako), anti‐CD86 (Abcam), or anti‐CD206 (Cell Signaling Technology). Finally, confocal laser scanning microscopy (Nikon, C2) was employed to observe and collect the corresponding images.

### Western Blot

The hippocampal tissues, as well as BV2 and primary microglial cells, were homogenized with the RIPA buffer (Beyotime Biotechnology) containing a protease inhibitor cocktail (APExBIO) and centrifuged. The supernatants were quantified with the BCA protein assay kit (Beyotime Biotechnology), then separated on SDS‐polyacrylamide gels, transferred to PVDF membranes (Millipore), blocked at 37 °C, and incubated with antibodies including APP (Cell Signaling Technology), Aβ1–42 (Cell Signaling Technology), Iba‐1 (Abcam), IL‐1β (Cell Signaling Technology), iNOS (Cell Signaling Technology), TNF‐α (Wanleibio), Arg‐1 (Abcam), IL‐10 (Wanleibio), TGF‐β (Cell Signaling Technology), HKI (Cell Signaling Technology), HKII (Abcam), PDK1 (Cell Signaling Technology), PDK2 (Abcam), and β‐actin (Cell Signaling Technology). After incubating with HRP‐conjugated secondary antibodies (EarthOx Life Science), the bands were visualized by the ECL Ultra Western HRP substrate (Millipore) and images were acquired by Chemiluminescence Gel Imaging System (Micro Chemi 4.2, DNR). The immunoblots were quantified with Image J software and target proteins were standardized with β‐actin as a protein loading control.

### Transmission Electron Microscope (TEM) Analysis

The hippocampal tissues or BV2 cells were fixed in ice‐cold 2.5% glutaraldehyde for 1 h, washed three times with sodium dimethyl arsenate buffer, and immersed in 1% osmium acid for 1 h. The samples were rinsed with double distilled water, dehydrated through 50% ethanol, 70% ethanol, a graded acetone series (80–100%), and embedded in Epon. Then, ultrathin sections (60 nm) were stained with uranium acetate and lead citrate successively and examined under a transmission electron microscope (Hitachi, H‐7650) at 12 000 or 30 000 magnification.

### Flow Cytometry for mtROS and Mitochondrial Membrane Potential Measurements

Flow cytometry for mtROS and mitochondrial membrane potential measurements was performed to assess the expression of mtROS and mitochondrial membrane potential measurements. Briefly, BV2 cells were treated with or without COR (1 µg mL^−1^) for 1 h, followed by stimulation with Rotenone+Antimycin A (ROT/AA, 0.5 µmol; Sigma–Aldrich), 2‐DG (50 mmol; Sigma–Aldrich) or WZB117 (10 µmol; MedChemExpress) for 24 h. Then, MitoSOX Red Mitochondrial (Thermo Fisher SCIENTIFIC) and Mitochondrial membrane potential assay kit with JC‐1 (Beyotime Biotechnology) experiments were carried out according to the manufacturer's instructions. Fluorescent measurements were immediately read using a FACSCalibur instrument (Becton‐Dickinson).

### Cell Viability Assay

The viability of the SH‐SY5Y and primary neurons was investigated by the Cell Counting Kit‐8 (CCK‐8 Kit; Dojindo) assay. Briefly, before the measurement, BV2 cells and rat primary microglial cells were seeded in 12‐well plates. After being pretreated without or with COR (1 µg mL^−1^) for 1 h, LPS (1 µg mL^−1^) for 1 h, and Aβ1‐42 (10 µmol) for 24 h, the cells were replaced with fresh media and culture for 24 h.

SH‐SY5Y cells or primary neurons were plated in 96‐well plates and grew overnight. Then, the supernatants were replaced with the conditioned media of BV2 cells or rat primary microglial cells. After incubation for 24 h, the CCK‐8 reagent was added to each well, and the absorbance at a wavelength of 450 nm was measured to test the cell viability with a multi‐mode reader (LD942).

### Measurement of Oxygen Consumption Rate (OCR) and Extracellular Acidification Rate (ECAR)

10^4^ BV2 or primary microglial cells were seeded into each well of 24‐well assay plates (Agilent Technologies) and grown overnight. OCR and ECAR were performed according to the manufacturer's protocol of the XF Cell Mito Stress Test Kit and XF Glycolysis Stress Test Kit (Agilent Technologies) using the Extracellular Flux Analyzer XF24.

### Mitochondrial Isolation

A mitochondrial extraction kit (Beyotime Biotechnology) was used to isolate the mitochondria of SH‐SY5Y cells, SVGP12 cells, and BV2 cells according to the manufacturer's protocol.

### Biotin‐COR(B‐COR) Synthesis

First, 74.316 mg 1‐hydroxybenzotriazole (HOBt; Solarbio LIFE SCIENCES) and 122.155 mg Biotin (Solarbio LIFE SCIENCES) and were mixed and dissolved in 1.96 mL N, N‐Dimethylformamide (DMF; Aladdin), followed by adding 115.02 mg EDCI (Shanghai yuanye Bio‐Technology) at room temperature for 1 h. Then, 125.625 mg cordycepin was added to the mixture at room temperature for 30 min. B‐COR powder was obtained by thin layer chromatography after the mixture was filtered.

### Cordycepin Pull‐Downs

Biotin‐cordycepin (B‐COR) was used in pull‐down experiments. LPS+Aβ1‐42‐induced BV2 cells were lysed on ice to obtain whole cell lysate or extract mitochondria using the Cell Mitochondria Isolation Kit (Beyotime Biotechnology). Pretreat the lysates or mitochondria with COR or B‐COR, then incubate with streptavidin magnetic beads (Thermo Fisher Scientific) at 4 °C overnight. The bead‐COR complexes were washed, eluted with sample buffer, and run on an SDS‐PAGE gel the following day. Whole lanes for each condition were cut for protein identification.

### Measurement of HK and PDH Activities

BV2 cells were treated without or with COR (1 µg mL^−1^) or/and 2‐DG (2, 5, 10 mmol) or/and VER‐246608 (40, 80, 100 nmol; MedChemExpress) for 1 h, followed by stimulation with LPS (1 µg mL^−1^) for 1 h and Aβ1‐42 (10 µmol) for 24 h. The corresponding HK and PDH levels were tested according to the kit instructions (Solarbio LIFE SCIENCES).

### Determination of Extracellular Lactate and Glucose Consumption

The BV2 cells were seeded in 12‐well plates, washed with PBS, and then incubated with KRPH buffer supplemented with 10 mmol glucose and 0.2% BSA for 2 h. Then, the supernatants were analyzed using the l‐Lactate Assay Kit (Cayman Chemical) and Glucose Colorimetric/Fluorometric Assay Kit (BioVision) according to the manufacturer's protocol.

### Statistical Analysis

All experimental data were performed at least three times and presented as mean ± SEM. Statistical differences between groups were assessed by Two‐way or One‐way analysis of variance (ANOVA), followed by a post hoc Turkey test using Prism (version 9.0.0). *p *< 0.05 was presented as statistically significant.

### Ethical Statement

All experimental animal procedures strictly complied with the Standard Medical Laboratory Animals' Care and Use Protocols Laboratory and the Animal Ethical Standards of China Medical University.

## Conflict of Interest

The authors declare no conflict of interest.

## Author Contributions

X.Z., S.G., and L.M. contributed equally to this work. M.Y.L. and M.J.W. conceived and designed this study. X.H.S. planned the experiments. X.Z., S.Q.G., W.F.Y., and K.D. carried out the detection in vivo. X.Z., L.H.M., W.J.J., Z.H.Y., and H.C.J. executed the experiments in vitro. J.H.T., J.R.D., and M.Y.L. performed the primary cell culture. L.C.J. and G.W.M. performed the GEO experiments. The chemical synthesis of B‐COR was conducted by J.W.L. and Y.Z.W. Moreover, B.B.W., X.J., and M.L.G performed the HPLC‐MS/MS experiments. S.Q.G. and L.H.M. conducted the statistical analysis of the results with the assistance of the other authors. X.Z., S.Q.G., and L.H.M. prepared the manuscript. M.Y.L., M.J.W, and X.H.S. reviewed and revised the manuscript. All authors discussed and approved the final version of the manuscript.

## Data Availability

The data that support the findings of this study are available from the corresponding author upon reasonable request.
